# BK Channels Reveal Novel Phosphate Sensitivity in SNr Neurons

**DOI:** 10.1371/journal.pone.0052148

**Published:** 2012-12-20

**Authors:** Juan Juan Ji, Lianwan Chen, Xuezhi Duan, Xueqin Song, Wenting Su, Peng Zhang, Li Li, Shuyun Bai, Yingchun Sun, Nobuya Inagaki

**Affiliations:** 1 State Key Laboratory of Brain and Cognitive Sciences, Institute of Biophysics, Chinese Academy of Sciences, Beijing, China; 2 Department of Physics, Northeast Normal University, Changchun, China; 3 Department of Diabetes and Clinical Nutrition, Graduate School of Medicine, Kyoto University, Sakyo-ku, Kyoto, Japan; Indiana University School of Medicine, United States of America

## Abstract

Whether large conductance Ca^2+^-activated potassium (BK) channels are present in the substantia nigra pars reticulata (SNr) is a matter of debate. Using the patch-clamp technique, we examined the functional expression of BK channels in neurons of the SNr and showed that the channels were activated or inhibited by internal high-energy phosphates (IHEPs) at positive and negative membrane potentials, respectively. SNr neurons showed membrane potential hyperpolarization under glucose-deprivation conditions which was attenuated by paxilline, a specific BK channel blocker. In addition, Fluo-3 fluorescence recording detected an increase in the level of internal free calcium ([Ca^2+^]_i_) during ischemic hyperpolarization. These results confirm that BK channels are present in SNr neurons and indicate that their unique IHEP sensitivity is requisite in neuronal ischemic responses. Bearing in mind that the K_ATP_ channel blocker tolbutamide also attenuated the hyperpolarization, we suggest that BK channels may play a protective role in the basal ganglia by modulating the excitability of SNr neurons along with K_ATP_ channels under ischemic stresses.

## Introduction

BK channels play important roles by couple multiple factors, such as intracellular Ca^2+^, phosphates and plasma membrane potential, to cellular activity and neurotransmitter or hormone release in various cells. BK channels are thought to be present and functional in various neurons of the central nervous system. They contribute to action potential repolarization in neostriatal cholinergic interneurons [1], afterhyperpolarization in hippocampal CA1 neurons [2], dendritic excitability in neocortical pyramidal neurons [3], and high-frequency firing in hippocampal pyramidal cells and neurons of the dorsal root ganglion [4], [5]. The involvement of BK channels in membrane hyperpolarization or alterations of output current caused by metabolic stress has been reported in striatal large aspiny interneurons [6], rat midbrain dopaminergic neurons [7], and neocortical neurons [8]. In addition, research on purkinje cells lacking BK channels has indirectly shown that they contribute to the afterhyperpolarization of the action potential, thus providing evidence of the involvement of BK channels in the excitability of purkinje cells [9]. This variety of functions suggests that BK channels from different sources have different cellular functions, and should be investigated serially in each new brain region in which they are present.

The response of BK channels to intracellular phosphates determines their involvement in the function of neurons and varies across different neurons. One type of potassium channel in substantia nigra pars compacta (SNc) neurons with a high conductance of 220 pS is activated by oxygen deprivation and inhibited by internal ATP, ADP, and AMP-PNP (a non-hydrolyzable ATP analog) at positive membrane potential [10]. Recently, we showed that BK channels are functional in SNc and LGP (lateral globus pallidus) neurons, and that they are inhibited by IHEPs at negative transmembrane potential, but they are not sensitive to AMP [11], [12]. BK channels have also been shown to be activated in the depression of LGP neurons caused by the depletion of intracellular ATP induced by the uncoupler carbonyl cyanide m-chlorophenylhydrazone (CCCP) [12]. However, intracellular ATP has also been shown to activate BK channels in CA1 and cortical neurons [13], [14]. These different response patterns to intracellular phosphates indicate that BK channels may play different roles under different metabolic conditions in different neurons, and thus to fully understand the cellular function of BK channels in different brain regions, the response pattern of these channels to intracellular phosphates must be clarified.

The SNr, the output nuclei of the basal ganglia, is the central gate controlling the propagation of seizures, and inactivity of its GABAergic neurons, the major neurons of the SNr, is believed to protect animals from generalized seizures caused by metabolic stresses such as hypoxia and hypoglycemia [15]. GABAergic neurons fire spontaneously at a high frequency in SNr [15], [16]. Generation of action potential and signal transduction requires constant input of ATP [17]. Thus, SNr neurons may have high energy requirements, and be vulnerable to ischemic conditions. In previous studies involving MTT-staining of brain tissues, we confirmed that mitochondrial redox response is dependent on oxygen contained in the MTT solution, and mitochondrial redox potential is significantly attenuated by transient deprivation of glucose and oxygen in the SNr [18], [19]. In addition, the substantia nigra itself readily lapses into ischemic conditions induced by infarction, since it is supplied by blood from branches of the posterior cerebral artery, which includes common sites of atherosclerosis and occlusion [20]. It would thus be interesting to investigate the role of BK channels in SNr neurons under energy-deprived conditions.

Existing molecular biological evidence for the expression of BK channels in the SNr is inconclusive. Positive evidence suggesting their existence includes immunoreactivity of the SNr for the α subunit (Slo1) [21] and β subunit [22] of BK channels. In addition, a putative BK channel gene, which shows high homology with the mouse *Slo* gene, has been cloned from a cDNA library of human substantia nigra by hybridization screening [23], and expression of β4 subunit mRNA has been found in the rat substantia nigra [24]. On the other hand, expression of *Slo1* mRNA has not been detected in rat SNr [21]. The expression of *Slo*1 mRNA in brain regions such as the ventromedial hypothalamic nucleus neurons [25], suprachiasmatic nucleus [26], SNc [11], and LGP [12], which are known to have functional BK channels, has also not been detected [21], [27]. Therefore further research is required to clarify and verify the presence and functional characteristics of BK channels in the SNr.

To understand the alteration of neuronal activity in the SNr under different metabolic conditions, i.e. ischemic conditions, we used cell physiological techniques and methods to investigate whether BK channels are functionally expressed in SNr GABAergic neurons. We found that BK channels are present in SNr neurons, are bidirectionally modulated by IHEPs, and are involved in the hyperpolarization of plasma membrane potential under glucose-deprived conditions.

## Results

### Characterization of GABAergic Neurons in Dissociated SNr Neurons

Neurons selected for electrophysiological and fluorescent experiments were spindle-like or polygonal in shape with a longitudinal diameter of 15–25 µm and a spontaneous firing rate greater than 1 Hz. SNr neurons (n = 294) from C57BL/6 mice, with a longitudinal diameter of 19.4±0.2 µm, membrane potential of −51.8±0.4 mV, firing rate of 10.4±0.4 Hz and firing half-width of 3.4±0.1 ms, were recorded in perforated whole-cell recording mode. Thirty-eight of the 294 neurons were stimulated by serially-stepped current pulses, and did not show hyperpolarization-activated currents (*I*_h_) at outputs of transmembrane potentials more positive than −120 mV (Figure S1). These basic membrane characteristics are similar to those reported for GABAergic neurons of SNr [15], [16], [28], [29].

### BK Channels are Present in SNr Neurons

**Figure 1:**
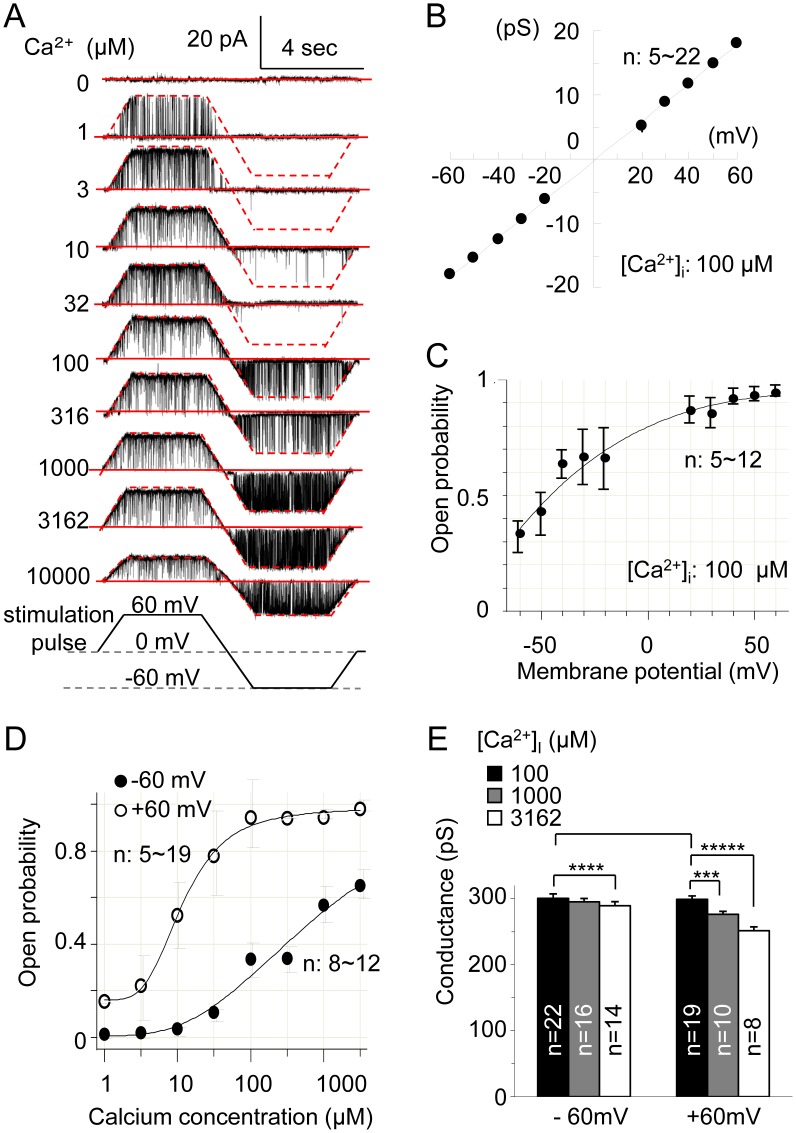
Characteristics of single BK channels in SNr neurons. (A) Representative recordings of single-channel currents at transmembrane potentials of −60 mV to 60 mV and [Ca^2+^]_i_ of 100 µM. The solid red line denotes the closed state and the dotted red line denotes the open state. (B) The relationship between single channel currents and holding membrane potential. The *I*–*V* curve was obtained by fitting a linear equation to the data. (C) The relationship between *P*_open_ and transmembrane potential. The *P*_open_-*V_m_* curve was obtained by fitting the Boltzmann equation to the data. (D) The relationship between *P*_open_ and [Ca^2+^]_i_. The *P*_open_-[Ca^2+^]_i_ curve was obtained by fitting the Hill equation to the data. (E) The effect of intracellular free calcium on conductance of the channel at membrane potentials of –60 mV and +60 mV. ^***^, *P*<0.005; ^****^, *P*<0.001; ^*****^, *P*<0.0001.

Firstly, to determine whether BK channels are present and functional in SNr neurons, we examined single channel currents by performing inside-out patch-clamp recording using symmetrical 140 mM [K^+^]_o_/[K^+^]_i_ solutions. The physiological and pharmacological properties of the channels were investigated at positive and negative transmembrane potentials by oscillating the holding membrane potential from −60 mV to +60 mV using pCLAMP9 software, and [Ca^2+^]_i_ was set at 100 µM to obtain moderate *P*_open_ BK channel currents at both positive- and negative- transmembrane potentials, except when testing their [Ca^2+^]_i_ dependency. BK channel currents were recorded in 44 of the 78 patches excised from isolated SNr neurons, and the average positive rate of channel currents was 2.8 current levels per patch in these patches. The open probability (*P*_open_) of the channel was dependent on [Ca^2+^]_i_ at both positive and negative transmembrane potential (Fig. 1). In almost all recordings, no notable decline in channel activity was observed even when the recording time was prolonged to 60 min.

### Properties of SNr Neuron BK Channels

At a physiological free-calcium concentration of 1 µM, the channel opened at a transmembrane potential of 0 to +60 mV, with a corresponding *P*_open_ value of 0 to 17%, and closed at negative potentials (Fig. 1a). This indicates that the main role of the BK channel is not to influence the plasma membrane potential under physiological conditions.

The *I–V* curve of the BK channel was almost a perfect linear, passing through the point (0, 0). According to the gradient of the *I–V* curve, the conductance of the BK channel was 301 pS (*R* ^2^ = 0.999; *n* = 8–22) between −60 ∼ +60 mV (Fig. 1a, b). *P*_open_ was dependent on transmembrane potential, and its half-maximal value occurred at −48 mV when the Boltzmann equation was fitted to the *P*_open_-voltage data (Fig. 1a, c). *P*_open_-[Ca^2+^]_i_ relationships were investigated at a range of 0 µM∼10 mM Ca^2+^ (Fig. 1a, d). EC_50_ (half-maximal effective concentration, *k*) values of 270 µM (*n* = 9–12) and 12 µM (*n* = 6–9) were obtained at transmembrane potentials of −60 mV and +60 mV respectively by fitting the Hill equation to *P*_open_-[Ca^2+^]_i_. When [Ca^2+^]_i_ was limited to not more than 1 mM, the conductance of the channel did not change. We observed that BK channel conductance decreased by 15% (*n* = 7) and 42% (*n* = 3) at +60 mV, and decreased by 0% and 18% (*n* = 3) at −60 mV, at extremely high [Ca^2+^]_i_ of 3 mM and 10 mM, respectively (Fig. 1a, e), although these decreases are likely not of physiological significance.

**Figure 2:**
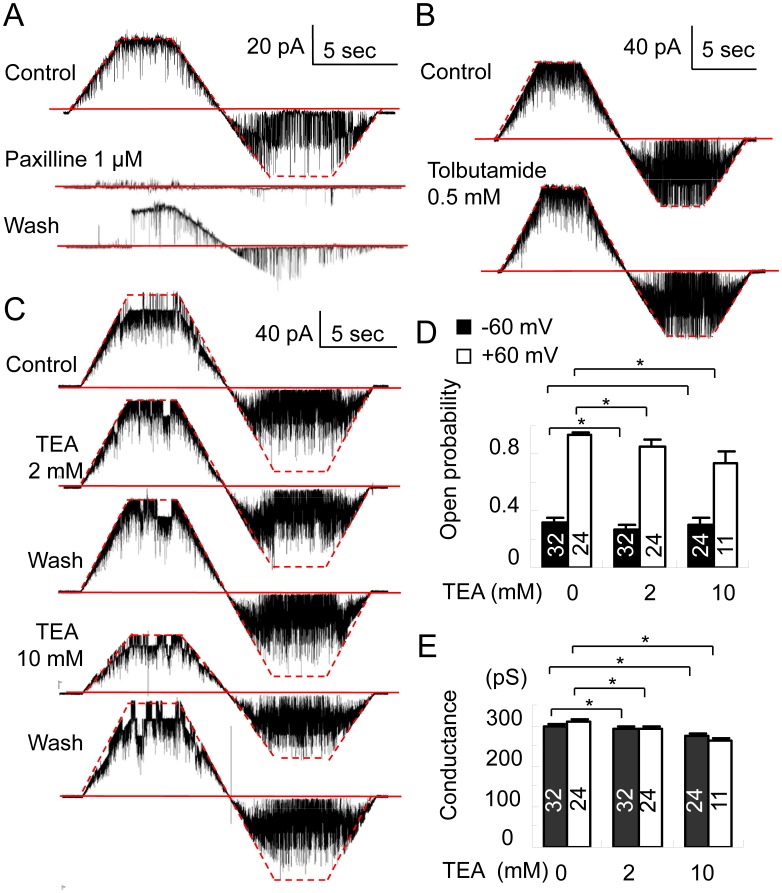
Effects of blockers on the activity and conductance of the BK channel. (A–C) Representative recordings of single-channel currents with or without internal paxilline (A), tolbutamide (B), and TEA (C). The solid red line denotes the closed state and the dotted red line denotes the open state at the second (A), fifth (B), and fourth (C) levels. (D–E) The effect of internal TEA on the open probability (D) and conductance (E) of the channel at membrane potentials of –60 mV and +60 mV. Internal free calcium (100 µM) was present throughout the experiment. Numbers in the columns represent the numbers of recorded patches. ^*^, *P*<0.05.

We next used potassium-channel blockers to confirm that these channels were indeed BK channels. Paxilline, a specific BK channel blocker, blocks BK channels both intra- and extracellularly [30]–[32]. Channel currents were completely blocked to zero open rate at both −60 mV and +60 mV by the internal application of 0.5 µM and 1 µM paxilline (*n* = 12) (Fig. 2a), and the effects were difficult to reverse. TEA, a non-specific blocker of voltage-dependent potassium channels, is often used to investigate the functional and structural characteristics of BK channels [33]. TEA at concentrations of 2 mM and 10 mM on the internal side of excised patches, decreased the K^+^ penetrability of BK channels by lowering *P*_open_ and conductance, and the extent of the decrease was dose-dependent (Fig. 2c–e). This decrease was especially prominent at positive voltages. The TEA sensitivity observed here was more conspicuous than that reported for BK channels in the hippocampus [34] and neocortical neurons [35] and similar to that reported for BK channels in SNc neurons [11] and the LGP neurons [12].

Tolbutamide (0.5 mM), a specific blocker of β-cell K_ATP_ channels, did not affect BK channel currents when the channels were opened by 100 µM Ca^2+^ at 0 mM ATP (8 patches) (Fig. 2b). This result indicates that it is possible to differentiate the cellular functions of BK and K_ATP_ channels, K_ATP_ channels being highly abundant and functional in SNr GABAergic neurons [15], [36]. These responses to channel blockers are consistent with present knowledge on BK channel characteristics.

### BK Channels are Bidirectionally Modulated by IHEPs

**Figure 3:**
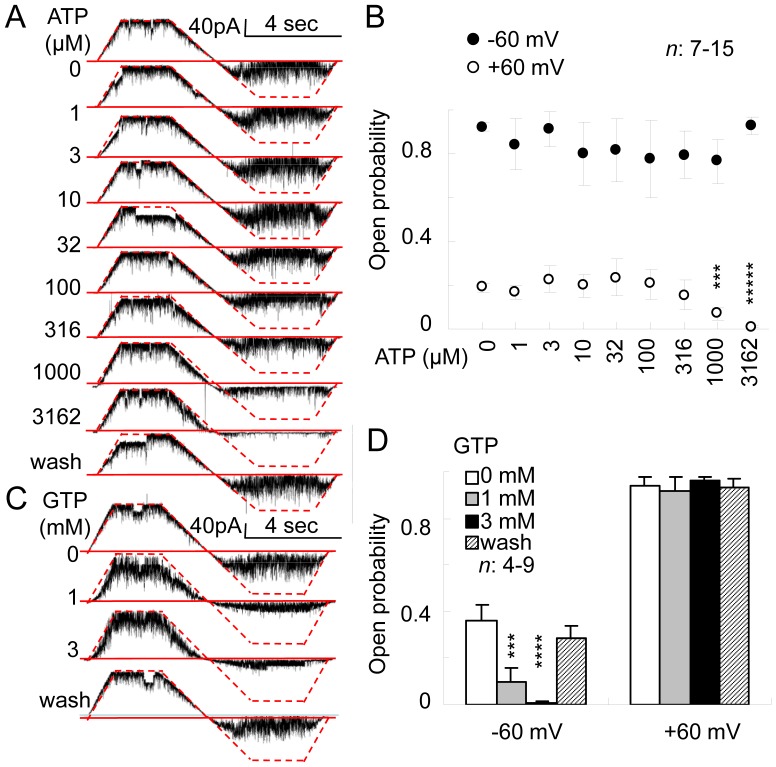
Sensitivity of BK channels in SNr neurons to high-energy phosphate compounds. (A,C) Representative recordings of single-channel currents with different concentrations of internal ATP and GTP. The solid red line denotes the closed state and the dotted red line denotes the open state at the fifth (A) and seventh (C) levels. (B) The relationship between the open probability and internal ATP concentration. (D) The effect of internal GTP on the open probability of the channel at holding membrane potentials of –60 mV and +60 mV. Asterisks above the data points indicate the statistical significance of comparisons with control groups. ^***^, *P*<0.005; ^*****^, *P*<0.0001.

To observe the effect of IHEPs on BK channels, phosphates were added to the inside of excised patches. ATP decreased the open probability of BK channels at negative potential in a dose-dependent manner, but had no significant effect at positive potential (Fig. 3A). Fitting of the Hill equation to *P*_open_ *-*ATP data at −60 mV, gave a Hill coefficient (*n*) of −1.68 and an *IC_50_* (half-maximal inhibitory concentration) of 0.65 mM (*n* = 15) (Fig. 3B). The effect of 1 mM and 3 mM GTP at both positive and negative membrane potential was similar to that of ATP at the same concentrations (*n* = 9), but 3 mM AMP did not have a visible effect on BK channel currents (*n* = 4). The conductance of BK channels was independent of internal phosphates at both positive and negative transmembrane potentials. These results suggest that IHEPs bidirectionally modulate the opening of the BK channels of SNr neurons.

### Intracellular ATP Levels Decrease after Glucose Deprivation

We examined the effect of glucose deprivation on changes in membrane potential in SNr neurons. The 159 SNr neurons exposed to glucose-free ACSF (artificial cerebrospinal fluid) could be divided into three groups based on changes in membrane potential: 17 (11%) were depolarized until the membrane potential disappeared, 28 (18%) were hyperpolarized after a slight depolarization, and 114 (71%) were directly hyperpolarized. Initial characteristics of these three response types are detailed in Figure S1. The latter two groups of neurons which showed ischemic hyperpolarization accounted for 90% of the 159 neurons and were selected for further evaluation. The neurons of these two groups hyperpolarized with max values of −9.3±0.5 mV and −12.0±1.3 mV (*P*<0.0005), respectively, 9.7±0.8 min and 12.2±1.9 min (*P*>0.05) after glucose-deprivation began. Tolbutamide was used to test for hyperpolarization-accompanied reductions in intracellular ATP, since pancreatic β-cell K_ATP_ channels are rapidly sensitive to intracellular ATP and are highly expressed in SNr neurons [36], and we have observed that tolbutamide attenuates the hyperpolarization induced by oxygen deprivation [15]. Here, 0.1 mM tolbutamide markedly attenuated the hyperpolarization induced by glucose deprivation (50/56). These results suggest that the intracellular ATP level was reduced by glucose deprivation and that this reduction can be detected by hyperpolarization. In line with the characteristics of single BK channels, this reduction in the level of intracellular ATP should result in the direct disinhibition of BK channels and their activation due to the dechelation of intracellular free calcium during glucose deprivation of SNr neurons.

### Intracellular Ca*^2+^* Levels Increase on Glucose Deprivation

**Figure 4:**
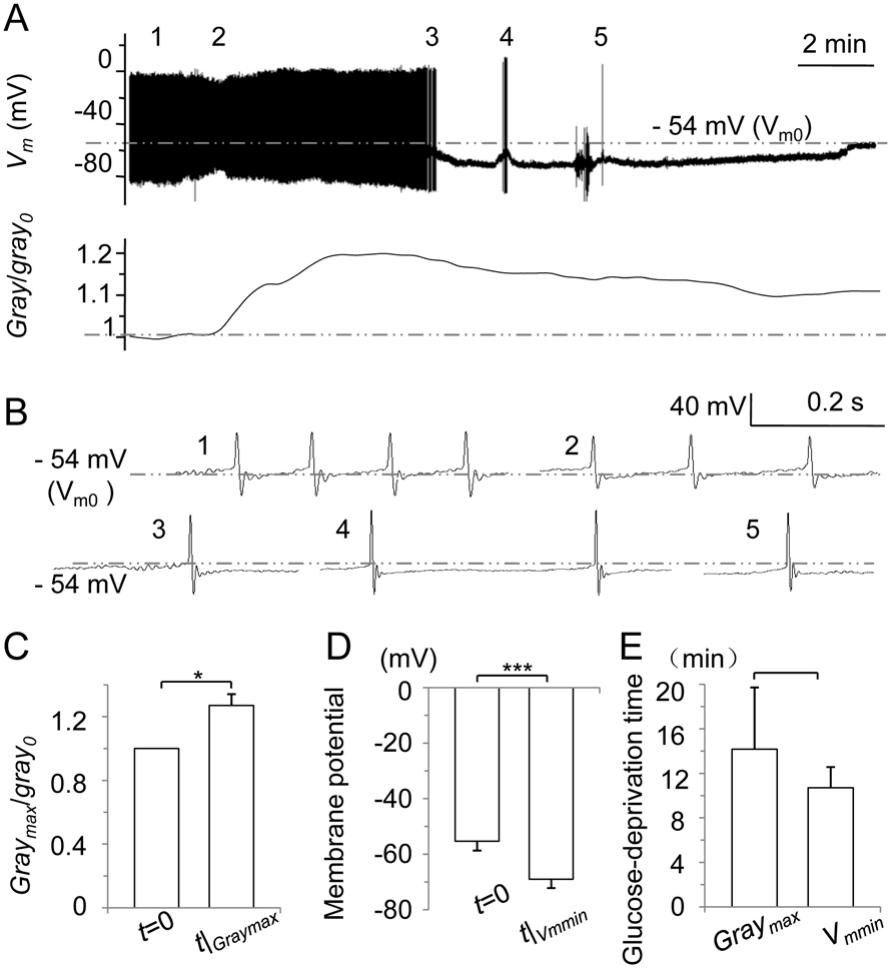
Glucose deprivation causes changes in membrane potential and rise in intracellular free calcium. (A) A co-recording of membrane potential (above) and calcium imaging (below). To increase the signal-to-noise ratio, we increased the initial sampling interval for calcium imaging from 20 sec to 1 min by averaging consecutive series of 3 points. (B) 1–5 represent expansions of the corresponding times in (A). (C–E) Histograms of normalized Gray (*Gray_max_*/*Gray_0_*) values (C), membrane potentials (D), and the time from the start of the glucose deprivation treatment (*t* = 0) to the maximum gray value *Gray_max_* and the minimum membrane potential *V_mmin_*, which corresponds to maximum hyperpolarization (E), at the time *t|_Vmmin_* or maximum gray value at the time *t|_Graymax_*. ^*^, *P*<0.05; ^***^, *P*<0.005.

To examine the involvement of intracellular free calcium in the cellular function of BK channels during glucose deprivation, experiments were performed in Fluo3-AM-loaded SNr neurons. Neurons (*n* = 13) were subjected to glucose deprivation treatment and membrane potential and fluorescence were co-recorded. A perforated whole-cell patch-clamp configuration was successfully maintained in only four of the neurons (Fig. 4), while a sharp increase in the action potential amplitude during electrophysiological recording of the remaining nine neurons indicated a switch to general whole-cell mode. All 13 neurons showed an increase in fluorescence during the hyperpolarization induced by glucose deprivation. Compared to perforated whole-cell patch-clamp recording alone, co-recording of membrane potential and fluorescence was less successful, possibly since the recording electrode oscillation originated from the mechanical shutter even though the shutter and microscope were connected by an optical fiber to reduce the transduction of the oscillation. A peak in gray value in the four neurons that maintained a perforated whole-cell patch-clamp configuration occurred at 10.7±1.9 min of glucose deprivation, almost co-incident with max hyperpolarization (14.2±5.6 min; *P*>0.05). The gray value increased by 32% (*P*<0.05) at max hyperpolarization (14.6±2.0 mV) relative to before the onset of glucose deprivation treatment. From these results we hypothesize that intracellular free calcium may contribute to the hyperpolarization caused by glucose deprivation injury by regulating BK channels.

### BK Channels are Involved in Cellular Responses to Glucose Deprivation

To investigate the role of BK channels in cellular responses to ischemic injury, SNr neurons were exposed to potassium channel blockers under control and glucose-deprivation conditions. The presence of 100 nM paxilline did not significantly alter the pattern of action potentials (4/4). 2 mM TEA increased the peak amplitude by 37% (*P*<0.01, *n* = 7) and the half-width by 61% (*P*<0.0001, *n* = 7), and decreased the firing rate by 33% (*P*<0.05, *n* = 7). Under glucose-deprivation conditions, paxilline attenuated the hyperpolarization in 10 out of 12 neurons. Charybdotoxin (4/5), a blocker of IK and BK channels, and TEA (44/49) attenuated the hyperpolarization to a similar extent as paxilline. Apamin, a small potassium channel blocker, did not affect the hyperpolarization (4/4). These results, in addition to our finding that the open probability of the channels is not higher than 17% when [Ca^2+^]_i_ is limited to the physiologically-relevant level of 1 µM (Fig. 1), indicate that BK channels may have only a negligible effect on the pattern of action potential generation, but contribute significantly to the membrane potential hyperpolarization induced by glucose deprivation. This is in contrast to other TEA-sensitive potassium channels which are functional when depolarized and are involved in action potential generation, but similar to the pancreatic β-cell type K_ATP_ channels found in SNr neurons that are known not to be involved in action potential generation but are involved in cellular responses to ischemic injury [15].

**Figure 5:**
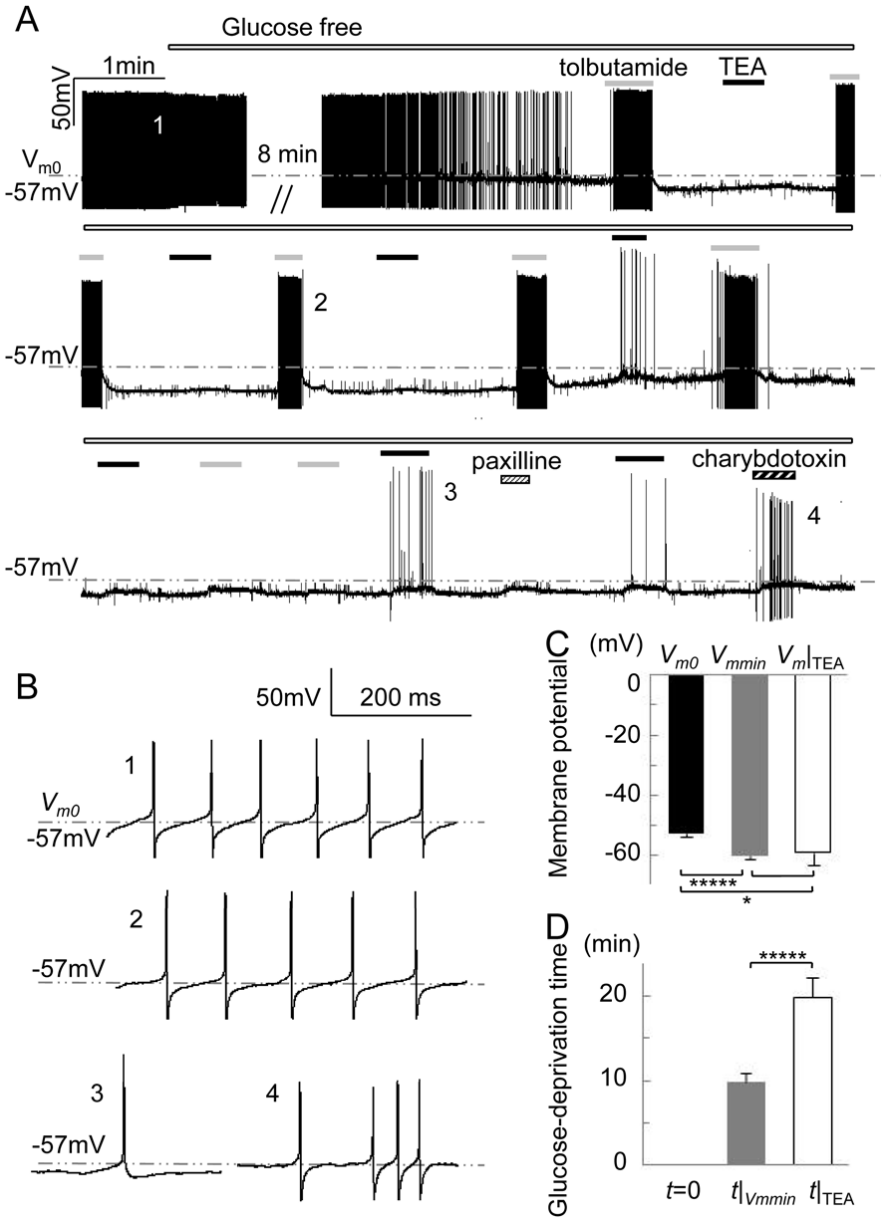
The effect of blockers on membrane potential hyperpolarization caused by glucose deprivation in SNr neurons. (A) A continuous recording of membrane potential. (B) 1–4 represent expansions of the corresponding times in (A). (C) Histogram of the membrane potential at the start of the glucose deprivation treatment (*V_m0_*; *t* = 0), minimum membrane potential (*V_mmin_*), which corresponds to maximum hyperpolarization, and the onset of the initial TEA effect (*V_m_*|*_TEA_*). (D) Time from the start of the glucose deprivation treatment (*t* = 0) to the onset of the minimum membrane potential corresponding to maximum hyperpolarization, or the initial TEA effect, at the time *t*|*_Vmmin_* or maximum gray value at the time t|_TEA_. ^*^, *P*<0.05; ^*****^, *P*<0.0001.

To discriminate the function of BK channels from that of K_ATP_ channels under glucose-deprivation conditions, 2 mM TEA and 0.1 mM tolbutamide were applied in succession repeatedly (Figure 5), after the glucose-deprivation-induced onset of hyperpolarization and termination of firing (*n* = 48). Here, TEA was used instead of paxilline, since it is easier to wash out from plasma membranes. The neurons had an initial resting potential of −48.9±0.8 mV and showed max hyperpolarization of 13.9±1.3 mV at 14.1±1.7 min after the onset of glucose deprivation. TEA and tolbutamide markedly attenuated hyperpolarization in 43/48 and 46/48 of neurons, respectively. The earliest attenuations caused by TEA were observed on average at 19.5±1.8 min of glucose deprivation, significantly later than max hyperpolarization (*P*<0.05), while attenuations caused by tolbutamide were observed from the onset of hyperpolarization. The effect of TEA did not decrease as glucose deprivation was prolonged, while tolbutamide lost its effect in 16/21 neurons within 60 min of glucose deprivation, on average at 24.8±3.4 min (*n* = 16) after the onset of glucose deprivation. When the first effects of TEA occurred, the membrane potential was −59.3±1.5 mV (*n* = 43), more positive than that at max hyperpolarization (−63.3±1.1 mV; *P*<0.0005) and more negative than that at resting potential (−54.7±0.8 mV; *P*<0.01). These results suggest that BK channels collaborate with K_ATP_ channels in the maintenance of glucose deprivation-induced hyperpolarization of plasma membrane potential; BK channels open after K_ATP_ channels and remain functional under conditions when K_ATP_ channels are no longer functional.

## Discussion

We report here that functional BK channels are present in the cell body of SNr GABAergic neurons. These channels are modulated bidirectionally by IHEPs at positive and negative transmembrane potentials. Under glucose-deprived conditions BK channels are activated by a decrease in intracellular ATP and an increase in intracellular Ca^2+^ and contribute to membrane potential hyperpolarization. The results presented here provide insights into how BK channels regulate SNr GABAergic neuron excitability by their unique response to intracellular IHEPs.

Concentrations of 1 mM and 3 mM ATP, close to physiological levels of intracellular ATP [17], in the internal solution of a membrane patch containing 100 µM Ca^2+^, should be able to chelate [Ca^2+^]_i_ to 34 µM and 13 µM, respectively, as calculated using Maxchelator (http://www.stanford.edu/~cpatton/maxc.html). At these calculated concentrations of free calcium, *P*_open_ should be 18% or 6% at −60 mV and 82% or 60% at +60 mV, respectively, according to the *P*_open_-[Ca^2+^]_i_ curve (Fig. 1). However, the experimental values of *P*_open_ at 1 mM and 3 mM internal ATP concentrations were 7% and 1% at −60 mV and 77% and 93% at +60 mV, respectively (Fig. 3). Thus, at positive membrane potential, the activation of the BK channel caused by ATP was sufficient to counterbalance the inhibition of the BK channel caused by the chelation of free calcium by ATP; at negative membrane potential, however, the effect of ATP on *P*_open_ was more inhibitory than would be expected, even bearing in mind the chelation of free calcium by ATP. The difference in the effects of ATP between positive and negative membrane potentials may be due to different structural features of the BK channel at positive and negative membrane potentials. The effect of GTP on BK channels was similar to that of ATP, but AMP had no effect on BK channel currents, suggesting that BK channels are bidirectionally modulated by intracellular high-energy phosphates in SNr neurons.

These effects of IHEPs may reflect differences in molecular structure between BK channels of the basal ganglia and other BK channels in the central nervous system. BK channels of the three basal ganglial nuclei, SNr, SNc, and LGP, are inhibited by IHEPs at negative-membrane potential [11], [12], and do not show contrasting responses to phosphate. Although some of the other CNS BK channels that have been reported also show similar responses as the BK channels in SNr neurons to some IHEPs, they do not respond to phosphates in the same way. In suprachiasmatic neurons, ATP decreases the open probability of a 260 pS BK channel at both negative and positive membrane potentials [26]. In neurons of the ventromedial hypothalamic nucleus, ATP decreases the open probability of 250 pS BK channels at outward currents (equivalent to positive-membrane potential) [25]. ATP inhibits the large K^+^ channels of human neocortical neurons which have a conductance of 180 pS, but these channels are also inhibited by AMP-PNP [35]. BK channels in rat cortical neurons can be activated by ATP, ADP, and GTP at positive membrane potential, but their activation requires the presence of Mg^2+^ [13]. That the BK channels from these three regions cannot be identified by the mRNA probes which have been used to identify BK channels in other regions of the rodent brain [21], [27] seems to suggest the molecular basis of SNr neuron BK channel sensitivity to IHEPs. In addition, iberiotoxin (100 nM), a widely-used specific BK channel blocker which can block BK channels in various neurons, did not show a clear effect under control (*n* = 5) or ischemic (*n* = 4) conditions in SNr neurons (data not shown). Moreover, BK channels of SNr neurons and LGP neurons [12], unlike most other reported BK channels, contribute to the maintenance of ischemic hyperpolarization but not to the action potential pattern. These results together support the hypothesis that basal ganglial BK channels may have a unique domain structure for sensing IHEPs.

Here, differences between BK channels in SNr neurons and those in the SNc and LGP were mainly in their [Ca^2+^]_i_ sensitivity. The EC_50_ of [Ca^2+^]_i_ for SNr BK channels was 12 µM at a membrane potential of +60 mV, larger than that for BK channels in the SNc and LGP, while at a membrane potential of −60 mV, the EC_50_ of [Ca^2+^]_i_ for SNr BK channels was 270 µM, lower than that for BK channels in the SNc and LGP. In addition, channel conductance was almost the same for inward and outward currents in the SNr BK channels, while BK channels in the SNc and LGP showed slight inward rectification. It is worth noting that the [Ca^2+^]_i_ and voltage dependence of BK channels in the SNr are lower and conductance is larger than in BK channels formed by slo 1 and β4 subunits [37], [38].

Taken together, these results indicate that functional BK channels, whose molecular structure may differ from BK channels in other brain regions, are present in basal ganglia; differences in the pattern of regulation between basal ganglial BK channels and BK channels in other brain regions suggest that modulation of basal ganglia output by the selective regulation of BK channels in basal ganglial neurons may be possible.

It is interesting that BK channels are involved in the maintenance of hyperpolarization induced by glucose deprivation. Figure S1 shows that glucose deprivation induced hyperpolarization was also observed in neurons lacking K_ATP_ channels (25/34) obtained from mutant mice which lacked the Kir6.2 subunit of the K_ATP_ channel [15], indicating that the BK channel can substitute for the K_ATP_ channel in initiating the hyperpolarization induced by glucose deprivation. In a previous report, we showed that the K_ATP_ channel of SNr neurons is opened by ATP depletion induced by oxygen-deprivation treatment, and plays a protective role in hypoxia-induced generalized seizure [15]. It is thus possible that BK channels also play a protective role under glucose deprivation, and possibly also under different types of ischemic injury, since a decrease in intracellular ATP level is also observed indirectly under oxygen-deprivation conditions in SNr neurons [15]. We previously demonstrated that after 15 min of oxygen-glucose deprivation at 31°C, the decrease in mitochondrial redox activity in adult mouse brain tissue is most severe in the LGP, substantia nigra (SN), and medial globus pallidus [18], [19]. Here, we also assessed mitochondrial redox activity in the SN after different treatments (Figure S2). 20 min of glucose deprivation did not cause a significant decrease in mitochondrial redox activity at room temperature. This is consistent with the fact that the level of ischemic injury is reduced by lowering the temperature. However, when 2 mM TEA was added to the glucose-deprived and control solutions, a significant decrease in mitochondrial redox activity was observed, but only in the former. This provides evidence for the protective role of BK channels under ischemic conditions.

The pathology of SNr has been highlighted in recent reports describing functional or histological injury in patients with basal ganglial diseases. Excessive activation of GABAergic output from the SNr in Parkinsonism is induced by reduced dopamine input to the striatum from the SNc [39]. Strategic infarct dementia is caused by single infarcts in functionally critical areas of the brain including the basal ganglia [40]. In addition, Parkinsonism due to cerebrovascular disease is a distinct clinicopathological entity, accounting for 4.4–12% of all cases of Parkinsonism [41]. Previous reports have suggested the involvement of BK channels in the function of the nervous system. For example, BK channel deficiency induces Purkinje cell dysfunction and cerebellar ataxia [9]. BK channels are also important targets for ethanol throughout the body [42]. Furthermore, the SNr BK channels presented here should be tried as a therapeutic target in epilepsy [43], because the SNr acts as a central gating system in the propagation of seizures and generalized seizures [15]. In this context, the BK channels in SNr neurons described here may become a potential target for selectively regulating the activity of SNr neurons due to their unique structural features. They are activated under ischemic stresses such as glucose deprivation and thus regulating them using BK channel openers such as NS1619 [32], [44], or altering levels of intracellular calcium or high-energy phosphates, may protect basal ganglial function and relieve symptoms in patients with strategic infarct dementia and Parkinsonism due to cerebrovascular disease. According to analyses on single channel currents (Fig. 1), BK channels are likely not involved in pattern of action potential under control conditions, it is thus possible that their regulation would have minimum adverse effects on the basal ganglial function. Consequently, the findings on the BK channels presented here could lead to a novel strategy for regulating the output of the basal ganglia and for protecting the function of the central motor control system by selective regulation of SNr BK channels.

## Materials and Methods

### Reagents

Papain, kynurenic acid, paxilline, trypsin, nystatin, Tris, ATP, GTP, Na_2_S_2_O_4_, CCCP, tolbutamide and tetraethylammonium (TEA), Flou-3 AM, and MTT were purchased from Sigma, USA. ADP and AMP were purchased from Amresco (USA). L-cysteine hydrochloride monohydrate was purchased from Wako (Japan). Deionized water (18 MΩ/cm) was obtained using a MILLIPORE-Q-B device (Millipore, USA) and used for all solutions.

### Sample Preparation

C57BL/6 mice were obtained from the Animal Experiment Center of Peking University Health Science Center (PR China). Mice lacking the Kir6.2 subunit of KATP channels (KO mice) were kindly provided by Prof. Susumu Seino (Kobe University) [15]. All experiments were approved by the Institutional Animal Administration Committee of the Institute of Biophysics of the Chinese Academy of Sciences (China) and Akita University Medical School (Japan).

Single neurons, obtained from P17-20 mice, enable rapid and complete alteration of extracellular conditions and were used in inside-out patch-clamp configuration and Fluo-3 fluorescence recording experiments. Brain slices, obtained from 13–14 week-old mice, maintained the living environment of synapses and cell bodies close to that *in vivo* and were used in MTT staining experiments (Supporting Information, Figure S2). The functional response of GABAergic receptors is already mature in rodents of these ages [45]. Brains were quickly removed from the craniums of decapitated mice and chilled for 1 min in ice-cold artificial cerebrospinal fluid (ACSF) containing (in mM): 145 NaCl, 4 KCl, 1 MgCl_2_, 2 CaCl_2_, 10 glucose, and 10 HEPES, pH 7.4. The pH value of experimental solutions was regulated with NaOH (for solutions used in the measurement of [Ca^2+^]_i_ changes) or Tris-hydroxymethylaminomethane (Tris) (all other solutions). ACSF was saturated with 100% O_2_ 30 min before use until the end of experiments. Coronal slices containing the rostral region of the SNr (thickness: 300 µm for cell dissociation or 130 µm for experiments using slices) were sectioned in ACSF solution with a vibroslicer (ZERO 1, Dosaka, Japan). The rostral region of the SNr was selected because it does not contain dopaminergic neurons, which are situated in the central region of the SNr [28], and because it is more vulnerable to ischemic injury than other regions of the SNr [19].

To obtain single SNr neurons, slices were incubated in an enzyme solution containing 26 units/ml papain, 1 mM kynurenic acid, and 1.5 mM L-cysteine hydrochloride monohydrate in ACSF, for 30 min at 30°C [15]. The central portion of the SNr area was cut free from the rest of the slice, and then mechanically dissociated with fire-polished glass pipettes in a poly-lysine-coated dish (Falcon, USA) under a microscope (CK30, Olympus, Japan) [11], [12], [15]. Cells were rehabilitated by perfusion with ACSF at 1 ml/min for at least 30 min and were used within 8 h. All recordings were carried out at 22–23°C using an inverted microscopy system (IX-70 microscope, Olympus, Japan; 1412M digital camera, DVC, USA; IPLab 3.6 software, Scanalytics, USA).

### Patch-clamp Recording

We obtained all data using an Axonpatch 200B patch-clamp amplifier (whose strength lies in single channel recording), and Digidata 1322 (Axon Instruments, USA), and data were analyzed with pCLAMP 9.0 (Axon Instruments). A low-pass filter of 2 kHz and a sampling frequency of 10 kHz were used. Single BK channel currents were recorded using an inside-out configuration, which enables rapid and complete alteration of the internal solution of the plasma membrane and is convenient for the investigation on the responses of ion channels to intracellular factors, in voltage-clamp mode (*V* = 0). Fire-polished pipettes were made from borosilicate glass capillaries using a puller (P-97, Sutter, USA) and polisher (2002-A, Yibo, China). Pipettes had a resistance of 3–4.5 MΩ when filled with pipette solution containing (in mM): 140 KCl, 0.1 CaCl_2_, 0.2 MgCl_2_, 10 HEPES, pH 7.2. The appropriate amount of CaCl_2_ was added to the pipette solution to make the bath solution. Beakers and tubes with good chemical resistance were used and 1 mM EGTA was used to remove the remaining Ca^2+^ in the perfusion tubes before measurement of calcium-dependence [11], [12]. Nystatin-perforated whole-cell recording, which is able to maintain intracellular conditions steady during the recording period [11], [12], [15], [46], was used to test the cellular functions of BK channels in current-clamp mode (*I* = 0) in isolated single cells. Pipettes had a resistance of 2–3 MΩ when filled with solution containing (in mM): 140 KCl, 10 HEPES, 50 µg/ml nystatin, pH 7.2. The bath solution used ACSF, or glucose/oxygen free ACSF. Bath solutions were perfused at 4–6 ml/min. Serial resistance and membrane capacitance were compensated as much as possible by the amplifier without producing an oscillation in the potential signal. Data were analyzed with Clampfit 9.0.

Reagents-containing solutions were directed to recorded patches or cells via a tube that was located at a distance of 3–5 mm. To ensure that the reagent concentration around the recording patch was accurate, the mixing of the reagent-containing solutions and bath solutions was prevented by regulating the rate of the reagent-containing solutions and making them to steady flow. Washout of the reagents was actualized by directing the control solution through the same tube till the recording signals were renewed. Ischemic conditions were mimicked by perfusion of an oxygen-glucose deprivation (OGD) solution containing (mM): 145 NaCl, 4 KCl, 1 MgCl_2_, 2 CaCl_2_, 2 Na_2_S_2_O_4_, and 10 HEPES, pH 7.4 which was saturated with 100% N_2_ from 30 min before use to the end of the experiments. To prepare a solution with a given free calcium concentration, we first prepared a solution with a high [Ca^2+^] of 100 mM and then diluted it to the final concentrations to minimize errors. The calcium-containing solutions were directed to recorded patches as the reagent-containing solutions were directed to.

### Fluorescent Ca^2+^ Imaging

Recovered cells were incubated for 20–30 min in ACSF containing 0.5–1 µM Fluo-3 acetoxymethyl ester (AM) in a culture dish placed in a container filled with oxygen and 100% humidity. Fluo-3 AM was dissolved in dimethyl sulfoxide to make a stock solution before diluting with ACSF, maintaining the final dimethyl sulfoxide concentration lower than 1‰. Cells were rehabilitated by ACSF perfusion for at least 30 min and then used for experiments. Ca^2+^ fluorescence was excited by a wavelength of 488 nm from a mercury lamp and was collected using a 520 nm filter [47]. During real-time recordings, excitations were for a period shorter than 0.5 sec with intervals of 20 sec, and were controlled using an electro-programmable shutter (VCM-D1, Uniblitz, USA). Images were acquired by the microscopy system described above and were analyzed using IPLab 3.6.

### Statistics

Data are presented as means ± standard error. The significance of differences between groups was calculated using the Student’s *t*-test. Differences were considered significant if *P*<0.05.

## Supporting Information

Figure S1
**Characteristics of dissociated SNr neurons during glucose deprivation treatment.** (A–C) Continuous recording of membrane potential in wild type (WT; a,b) and knock-out (KO; c) neurons. The responses to glucose deprivation in (A), (B), and (C) are type 2 (directly hyperpolarized), type 3 (hyperpolarized after a slight depolarization), and type 2 responses, respectively. (D) Representative pattern of membrane-potential responses to serially-stepped pulses. Traces correspond to the section in (A) marked by “
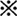
”. (E) Characteristics of WT and KO neurons with different responses to glucose deprivation; long diameter of the cell body, the half-width of firing, firing rate, and resting membrane potential. Data were obtained under control conditions before the onset of the glucose-deprivation treatment. (F) Histogram showing that the change in resting membrane potential in type 2 and type 3 neurons at the minimum membrane potential (*V_mmin_*) corresponds to the maximum hyperpolarization and that the maximum membrane potential (*V_mmax_*) corresponds to the maximum depolarization. (G) Histogram showing the time from the start of the glucose deprivation treatment (*t* = 0) to *V_mmin_* and *V_mmax_*. ^*^, *P*<0.05; ^**^, *P*<0.01; ^***^, *P*<0.005; ^****^, *P*<0.001; ^*****^, *P*<0.0001. All 159 WT neurons had an average longitudinal cell body diameter, resting potential, firing rate, and half-width of about 19.2±0.4 µm, −52.9±0.5 mV, 10.8±0.6 Hz, and 3.1±0.1 ms, respectively, and the 34 KO neurons had an average longitudinal cell body diameter, resting potential, firing rate, and half-width of about 20.1±1.2 µm, −50.0±0.9 mV, 10.3±1.7 Hz, and 4.2±0.3 ms, respectively. *P* values for the comparison of these characteristics between WT and KO neurons were determined using the Student’s *t-*test and were 0.175, 0.0071, 0.4188, and 0.0018, respectively. These electrophysiological characters of WT and KO neurons are similar to results from our previous report [15].(TIF)

Figure S2 **The effect of 2 mM TEA on the substantia nigra (SN).** Serial coronal slices (130 µm) containing the SN, indicated by closed curves, from C57BL/6 mice (d15-20) were prepared using a vibroslicer (ZERO 1, Dosaka) in ice-cold, O_2_-saturated artificial cerebrospinal fluid (ACSF) containing the following (in mM): 150 NaCl, 5 KCl, 1 MgCl_2_, 2 CaCl_2_, 10 glucose, and 10 HEPES, pH 7.4 (Tris). Slices were then incubated for 30 min in ASCF in multiple perfusion chambers at room temperature (23–24°C) for recovery. Each 1.5 ml chamber had a perfusion rate of 4.0±0.2 ml/min and contained 1–2 slices. Here, the environment of the tissue could be regulated by exchanging the perfusion solution. The perfusion solution was changed to ACSF alone, ACSF without glucose or without glucose and oxygen, or to these solutions containing 2 mM TEA. Tissues were then incubated in MTT solution (i.e. 0.5 mg/ml MTT dissolved in ACSF) for 20 min followed by fixing with 4% paraformaldehyde for 2 hours, washing in phosphate buffer, mounting on a glass slide, and drying overnight [18], [19]. Images of the slices were acquired using a microscope (BX51WI, Olympus) equipped with a digital camera (1412M, DVC) and image acquisition software (IPLab 3.6, Scanalytics). Image analysis was conducted using IPLab 3.6 software (Scanalytics). Data are presented as means ± standard error (Data for single slices are the average values of the SNr from both sides). (A) Representative images of the SN. Slices form rostral to caudal serial coronal slices are displayed vertically. (B) Statistical results of the mitochondrial redox potential in the SN region. Asterisks above data points indicate the statistical significance of comparisons with the control group. ^**^, *P*<0.01; ^*****^, *P*<0.0001.
(TIF)
