# Supplementary figures and images for: BK Channels Reveal Novel Phosphate Sensitivity in SNr Neurons
Source: PLoS One. 2012 Dec 20;7(12):e52148. doi: 10.1371/journal.pone.0052148 (PMC3527394; doi:10.1371/journal.pone.0052148)

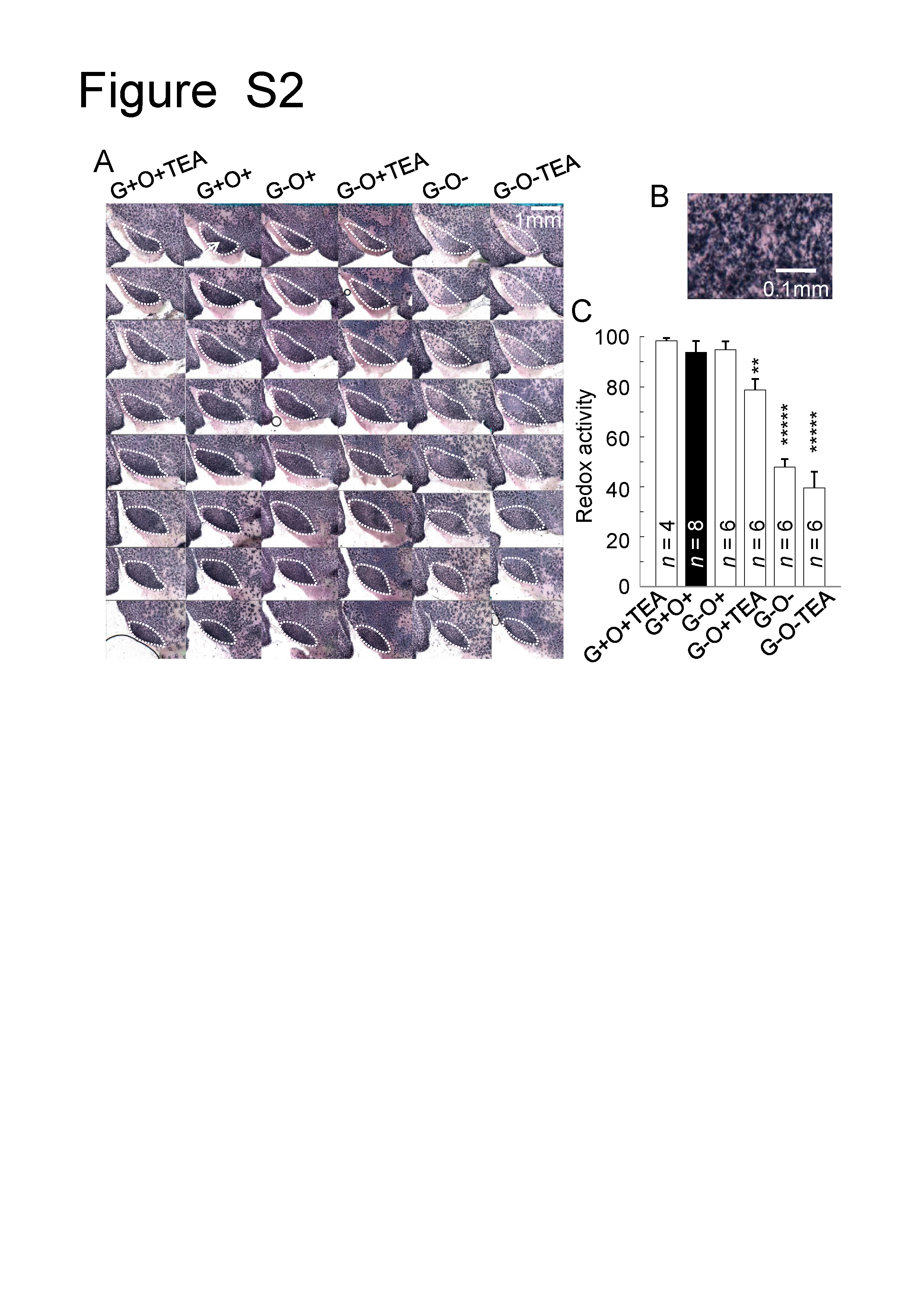

Supplement: Figure S2 — The effect of 2 mM TEA on the substantia nigra (SN). Serial coronal slices (130 µm) containing the SN, indicated by closed curves, from C57BL/6 mice (d15-20) were prepared using a vibroslicer (ZERO 1, Dosaka) in ice-cold, O2-saturated artificial cerebrospinal fluid (ACSF) containing the following (in mM): 150 NaCl, 5 KCl, 1 MgCl2, 2 CaCl2, 10 glucose, and 10 HEPES, pH 7.4 (Tris). Slices were then incubated for 30 min in ASCF in multiple perfusion chambers at room temperature (23–24°C) for recovery. Each 1.5 ml chamber had a perfusion rate of 4.0±0.2 ml/min and contained 1–2 slices. Here, the environment of the tissue could be regulated by exchanging the perfusion solution. The perfusion solution was changed to ACSF alone, ACSF without glucose or without glucose and oxygen, or to these solutions containing 2 mM TEA. Tissues were then incubated in MTT solution (i.e. 0.5 mg/ml MTT dissolved in ACSF) for 20 min followed by fixing with 4% paraformaldehyde for 2 hours, washing in phosphate buffer, mounting on a glass slide, and drying overnight [18], [19]. Images of the slices were acquired using a microscope (BX51WI, Olympus) equipped with a digital camera (1412M, DVC) and image acquisition software (IPLab 3.6, Scanalytics). Image analysis was conducted using IPLab 3.6 software (Scanalytics). Data are presented as means ± standard error (Data for single slices are the average values of the SNr from both sides). (A) Representative images of the SN. Slices form rostral to caudal serial coronal slices are displayed vertically. (B) Statistical results of the mitochondrial redox potential in the SN region. Asterisks above data points indicate the statistical significance of comparisons with the control group. **, P<0.01; *****, P<0.0001. (TIF) [file pone.0052148.s002.tif]
